# Interrater reliability in the application of the VPAS-PB: analysis among experienced judges

**DOI:** 10.1590/2317-1782/e20250169en

**Published:** 2026-04-17

**Authors:** Renata Christina Vieira

**Affiliations:** 1 Hospital Central Aristarcho Pessoa, Corpo de Bombeiros Militar do Estado do Rio de Janeiro – CBMERJ - Rio de Janeiro (RJ), Brasil.

**Keywords:** Voice, Voice Quality, Speech Perception, Auditory Perception, Phonetics

## Abstract

**Purpose:**

This study analyzes the interrater reliability of the Brazilian Portuguese version of the Vocal Profile Analysis Scheme (VPAS-PB) applied by experienced judges.

**Methods:**

Ten adult participants with no vocal or hearing complaints produced a standard sentence and two personal stories, which were recorded and later evaluated independently by three speech-language-hearing pathologists trained in phonetics and experienced with the protocol. Interrater reliability was estimated using internal consistency analysis (Cronbach’s Alpha).

**Results:**

Analysis with Cronbach’s alpha indicated good to excellent internal consistency for most vocal settings, with lower agreement for “lip spreading” and “falsetto.”

**Conclusion:**

This study demonstrates that the VPAS-PB has high interrater reliability and excellent internal consistency across most vocal settings, reinforcing its applicability in the Brazilian Portuguese context.

## INTRODUCTION

Auditory-perceptual evaluation of voice is widely recognized as a consolidated step in vocal quality analysis. However, its subjective nature poses challenges that require structured protocols capable of minimizing interrater variability and guiding systematic descriptions of observed vocal adjustments. The Vocal Profile Analysis Scheme (VPAS) stands out among these protocols, developed to systematize voice description based on the phonetic model of vocal quality^(1)^.

The phonetic model of vocal quality proposes describing the voice based on the integrated observation of auditory, acoustic, physiological, and articulatory cues, reflecting the different configurations and functions of the vocal tract during speech. VPAS-PB (Brazilian Portuguese version) is based on the principle of combined vocal adjustments, allowing for the simultaneous recording of different vocal production characteristics. This approach seeks to reflect the real complexity of vocal configurations and increase the descriptive accuracy of perceptual analysis.

The VPAS was adapted to Brazilian Portuguese^(2)^, maintaining the original theoretical structure but incorporating terminological adjustments that reflected the linguistic and cultural reality of Brazil. The adaptation considered advances in phonetics and speech physiology, especially regarding phonatory aspects. Terms such as breathiness and whisper, for example, were revised to ensure equivalence with greater semantic accuracy: whispery voice came to be associated with the breathy voice, while breathy voice corresponds to the murmured voice, although the latter term, common in descriptive phonetics, was not included in the final translation.

Previous national research has investigated the validity of applying VPAS, demonstrating the importance of specific training for raters to achieve adequate levels of consensus^(3)^. Accurate identification of vocal adjustments depends on both technical training and conceptual clarity of the parameters that make up the protocol.

International and national studies reinforce the importance of adopting structured protocols based on the phonetic model of vocal quality to reduce interrater variability and strengthen analysis reliability^(4,5)^. Clear phonetic references favor the consistent evaluation of vocal quality.

Moreover, factors such as the technical quality of audio samples (including adequate sampling frequency and noise control) are determinants for the accuracy of auditory-perceptual evaluation^(6)^.

Specific training of judges also exerts a significant influence. Studies indicate that structured training programs increase interrater agreement^(7)^, although scoping reviews point to the lack of consensus on the best method for training these professionals^(8)^.

The literature frequently uses the intraclass correlation coefficient (ICC) to measure interrater reliability^(9)^, especially in contexts of continuous data analysis. This study used Cronbach's Alpha, which is suitable for verifying the internal consistency of categorical judgments applied in the VPAS-PB.

The need for objective and reproducible assessments in forensics^(6)^ makes the adoption of formalized protocols even more critical. Thus, VPAS-PB offers an assessment method based on the phonetic model of vocal quality, favoring systematic and reproducible descriptions of vocal quality.

Controlling sociolinguistic, phonetic, and vocal variables has been indicated as relevant for the validity of auditory-perceptual evaluation^(4)^ and was incorporated into the method of this study.

Finally, comparative investigations indicate that the complete VPAS version allows for more detailed and consistent descriptions than simplified versions^(10)^. These findings reinforce the need to expand the teaching and application of the phonetic model of vocal quality by using VPAS to strengthen the training of evaluators and the quality of analyses in Portuguese.

Thus, this study aimed to analyze the internal consistency of auditory-perceptual evaluations performed with VPAS-PB, contributing to the methodological strengthening of vocal quality analyses in Brazilian Portuguese.

## METHODS

This is a descriptive observational study, approved by the institution's Research Ethics Committee (approval 2.153.565). All participants signed an informed consent form before data collection.

The study included 10 adults, native Brazilian Portuguese speakers, aged 34 to 49 years, residing in the same city. The choice of male subjects aimed to control for sex-related acoustic variables, avoiding the need to form two distinct groups. The researcher screened their voices through self-report and auditory-perceptual clinical listening to exclude participants with evident vocal changes. People with auditory complaints, a history of occupational voice use, and/or reading difficulties were also excluded. Each participant was instructed to make three speech sample recordings: reading a standard sentence and two spontaneous narrative accounts, in which they should share personal stories of emotional impact. Each spontaneous narrative lasted 1 minute on average.

The recordings were made in a controlled environment using a Zoom H5 recorder with a sampling frequency of 44,100 Hz, 16-bit resolution, and .wav file extension. The subjects were positioned 60 centimeters from the microphone to standardize the sound signal capture.

The samples were organized in collection format using Praat free software. Each set of samples was stored in individual folders, including the respective VPAS-PB for raters to fill out. The material sent to the judges also included standardized guidelines.

Three evaluators participated in the auditory-perceptual evaluation. All had training in phonetics and a minimum of 4 years of experience in applying VPAS-PB (Annex A). Each judge analyzed the samples independently, completing the protocol for each set of speech samples individually. The internal consistency analysis was based on these individual judgments.

The data resulting from the evaluation were organized into Microsoft Excel spreadsheets and then subjected to statistical analysis. The interrater internal consistency index was calculated with Cronbach's Alpha, using IBM SPSS Statistics software, version 23.0. The significance level was set at 5% (p < 0.05).

## RESULTS

Internal consistency analysis revealed high levels of interrater reliability for most vocal adjustments. Most Cronbach's alpha values ​​were above 0.75, indicating good to excellent consistency. Specific adjustments had only satisfactory or unsatisfactory scores, as detailed in Table 1.

**Table 1:** Interrater internal consistency: Cronbach's alpha of vocal adjustments

Vocal Adjustment	Cronbach’s Alpha (α)	Significance (p)	Reliability Status
Lip rounding	0.667	0.023	Satisfactory
Lip stretching	0.585	0.053	Unsatisfactory
Labiodentalization	1.000	< 0.001	High
Reduced lip extension	0.892	< 0.001	High
Increased lip extension	0.893	< 0.001	High
Closed jaw	1.000	< 0.001	High
Open jaw	0.956	< 0.001	High
Reduced jaw extension	0.914	< 0.001	High
Increased jaw extension	0.881	< 0.001	High
Advanced tongue tip	0.901	< 0.001	High
Retracted tongue tip	0.750	0.006	High
Advanced tongue body	0.869	< 0.001	High
Retracted tongue body	0.654	0.026	Satisfactory
Elevated tongue body	0.843	< 0.001	High
Lowered tongue body	0.903	< 0.001	High
Reduced tongue body extension	0.686	0.018	Satisfactory
Pharyngeal constriction	0.747	0.006	High
Pharyngeal expansion	0.960	< 0.001	High
Audible nasal escape	1.000	< 0.001	High
Nasalization	1.000	< 0.001	High
Denasalization	1.000	< 0.001	High
Elevated larynx	0.853	< 0.001	High
Lowered larynx	0.780	0.003	High
Tract hyperfunction	0.862	< 0.001	High
Tract hypofunction	1.000	< 0.001	High
Laryngeal hyperfunction	0.956	< 0.001	High
Falsetto	0.585	0.053	Unsatisfactory
Crepitation	1.000	< 0.001	High
Crepitating voice	0.852	< 0.001	High
Air escape	1.000	0.001	High
Breathy voice	0.667	0.023	Satisfactory
Raspy voice	0.932	< 0.001	High
Elevated usual pitch	0.821	0.001	High
Lowered usual pitch	1.000	< 0.001	High
Reduced pitch extension	0.750	0.006	High
Reduced pitch variability	0.646	0.029	Satisfactory
Increased pitch variability	1.000	< 0.001	High
Increased usual loudness	1.000	< 0.001	High
Reduced usual loudness	0.926	< 0.001	High
Increased loudness extension	1.000	< 0.001	High
Reduced loudness extension	1.000	< 0.001	High
Increased loudness variability	1.000	0.001	High
Reduced loudness variability	1.000	< 0.001	High
Interrupted continuity	1.000	< 0.001	High
Rapid speech rate	0.892	< 0.001	High
Slow speech rate	1.000	< 0.001	High
Adequate respiratory support	1.000	< 0.001	High
Inadequate respiratory support	1.000	< 0.001	High

**Caption:** α = Cronbach’s alpha. Statistical test used: internal consistency analysis

## DISCUSSION

The study results showed that applying VPAS-PB in auditory-perceptual evaluation of vocal quality generated high levels of internal interrater consistency, indicating the reliability of the protocol in Brazilian Portuguese. Cronbach's Alpha revealed high values, as most vocal adjustments had very good consistency, as detailed in Table 1.

Consistency was particularly high for laryngeal and supraglottic tension adjustments, with Cronbach's Alpha values ​​greater than 0.90. This reflects how clearly raters perceive these adjustments. The phonetic model of vocal quality facilitates the identification of adjustments that are more evident in speech^(1-3,10)^, which contributes to the high reliability observed in these cases.

On the other hand, some adjustments, such as rounded lips, retracted tongue body, and decreased pitch variability, had only satisfactory internal consistency (Cronbach's Alpha between 0.60 and 0.70). These adjustments involve more subtle and difficult-to-identify characteristics; hence, they are consistent with the literature, which points to greater perceptual variation in parameters of greater auditory salience^(3,11)^.

Two adjustments (lip stretching and falsetto) had unsatisfactory internal consistency (Cronbach's Alpha below 0.60). This indicates greater interrater variability, possibly due to the difficulty in identifying and classifying these adjustments consistently. The literature also highlights that subtle adjustments or those with low perceptual salience tend to have lower interrater reliability^(5)^.

The results demonstrated less agreement in adjustments related to more subtle or difficult-to-discriminate characteristics, such as lip stretching and falsetto, despite the robustness of VPAS-PB. A possible explanation is that such adjustments may have been little used by the participants or not sufficiently marked to be clearly perceived. Moreover, since they are male voices, certain adjustments may have been avoided or little explored. A previous study identified differences in the use of vocal adjustments between men and women^(12)^. Furthermore, sociolinguistic factors may have contributed to the lower occurrence of these adjustments, making their detection more difficult.

This study focused on the internal consistency of the assessments, an important aspect for verifying the reproducibility of the protocol in different contexts and with different raters. The use of Cronbach's Alpha was adequate to measure the reliability of the assessments, since the objective was not to measure intersubject variability, but rather the internal homogeneity of the assessments.

The findings also indicate that, despite the good overall reliability, training raters continuously is a decisive factor in improving consistency in judging adjustments of lower perceptual salience. Recent studies have pointed to the importance of more detailed training programs in auditory-perceptual evaluation^(8)^, especially in light of discussions about the supposed subjectivity of this type of analysis^(13)^. Despite criticisms of the accuracy of analyses based exclusively on auditory perception, the idea that only instrumental methods would be objective has been questioned, since their application involves human decisions and, therefore, is equally subject to variability^(11)^.

Besides training, the high reliability observed in this study may also be related to the raters' prior experience and the continuous VPAS-PB use over time. Familiarity with the protocol, developed through its recurrent practical application, may have favored the stability of judgments, especially in the more subtle adjustments^(3)^. This factor, although not directly measured, should be considered in future investigations on the impact of accumulated experience on perceptual reliability.

Despite the wide applicability of VPAS-PB in clinical and research settings^(14,15)^, previous studies have mostly focused on restricted groups regarding sex, age range, and linguistic variety^(12)^. Most investigations involve adult female speakers, often from the same regional and sociolinguistic background, which may limit the generalization of the findings. Thus, future research should apply the protocol to more diverse populations, including different genders, age ranges, regional accents, and languages, to verify whether they influence the perception and occurrence of the adjustments, helping to expand the ecological validity of the instrument, including its use in forensic examinations^(10,11)^.

Accurate auditory-perceptual evaluations are especially relevant in forensics. VPAS-PB offers a robust approach to ensure detailed and consistent descriptions of vocal quality, minimizing subjectivity in assessments^(10)^. This reinforces the effectiveness of the phonetic model of vocal quality adapted to Brazilian Portuguese.

## CONCLUSION

This study demonstrated that VPAS-PB has high interrater reliability, with excellent internal consistency in most vocal adjustments. These findings reinforce its applicability in the context of Brazilian Portuguese. However, adjustments with less perceptual salience, such as lip stretching and falsetto, revealed less agreement, pointing to the need to train raters continuously.

Cronbach's Alpha effectively assessed the homogeneity of judgments, and the results suggest that familiarity with the protocol may favor the stability of the analyses.

Future studies should expand sample diversity and investigate the impact of sociolinguistic, phonetic, and contextual variables, including applications in expert assessments. VPAS-PB is a promising systematic tool for reliable and replicable vocal assessments.

## Annex A. Vocal Profile Analysis Scheme – Brazilian Portuguese version (VPAS-PB)

**Table:**

	**FIRST PASS**		**SECOND PASS**									
	**Neutral**	**Nonneutral**					**Moderate**			**Extreme**		
							**1**	**2**	**3**	**4**	**5**	**6**
**1. Lips**	**Neutral**	**Nonneutral**	**Protracted**									
			**Stretched**									
			**Labiodentalization**									
			**Reduced extension**									
			**Increased extension**									
**2. Jaw**	**Neutral**	**Nonneutral**	**Closed**									
			**Open**									
			**Protracted**									
			**Reduced extension**									
			**Increased extension**									
**3. Tongue tip/side**	**Neutral**	**Nonneutral**	**Advanced**									
			**Retracted**									
**4. Tongue body**	**Neutral**	**Nonneutral**	**Advanced**									
			**Retracted**									
			**Elevated**									
			**Lowered**									
			**Reduced extension**									
			**Increased extension**									
**5. Pharynx**	**Neutral**	**Nonneutral**	**Constricted**									
			**Expanded**									
**6. Velopharynx**	**Neutral**	**Nonneutral**	**Audible nasal escape**									
			**Nasalization**									
			**Denasalization**									
**7. Larynx height**	**Neutral**	**Nonneutral**	**Elevated**									
			**Lowered**									
**B. GENERAL MUSCLE TENSION**												
**8. Vocal tract tension**	**Neutral**	**Nonneutral**	**Hyperfunction**									
			**Hypofunction**									
**9. Laryngeal tension**	**Neutral**	**Nonneutral**	**Hyperfunction**									
			**Hypofunction**									
**C. PHONATORY ELEMENTS**												
	**ADJUSTMENT**			**Present**			**Scale Degrees**					
				**Neutral**		**Nonneutral**	**Moderate**			**Extreme**		
							**1**	**2**	**3**	**1**	**2**	**3**
**10. Phonation mode**	**Modal**											
	**Falsetto**											
	**Crepitation/vocal fry**											
	**Crepitating voice**											
**11. Laryngeal friction**	**Air escape**											
	**Breathy voice**											
**12. Laryngeal irregularity**	**Raspy voice**											
**VOCAL DYNAMICS**		**Neutral**	**ADJUSTMENT**				**Moderate**			**Extreme**		
							**1**	**2**	**3**	**1**	**2**	**3**
**D. PROSODIC ELEMENTS**												
**13. Pitch (f0)**		**Usual**	**Reduced**									
			**Increased**									
		**Extension**	**Reduced**									
			**Increased**									
		**Variability**	**Reduced**									
			**Increased**									
**14. Loudness**		**Usual**	**Reduced**									
			**Increased**									
		**Extension**	**Reduced**									
			**Increased**									
		**Variability**	**Reduced**									
			**Increased**									
**15. TIME**												
**Continuity**					**Interrupted**							
**Speech rate**					**Rapid**							
					**Slow**							
**16. OTHER ELEMENTS**												
**Respiratory Support**					**Adequate**							
					**Inadequate**							

**Caption:** Vocal Profile Analysis Scheme (adapted to Brazilian Portuguese by Camargo and Madureira, 2008).
