# Supplementary material for: Interrater reliability in the application of the VPAS-PB: analysis among experienced judges
Source: Codas. 2026 Apr 17;38(3):e20250169. doi: 10.1590/2317-1782/e20250169en (PMC13142255; doi:10.1590/2317-1782/e20250169en)
Supplement: Translation

# Translation: Confiabilidade interavaliadores na aplicação do VPAS-PB: análise entre juízes experientes

**DOI:** 10.1590/2317-1782/e20250169pt

**Authors:** Renata Christina Vieira

**Affiliations:** 1 Hospital Central Aristarcho Pessoa, Corpo de Bombeiros Militar do Estado do Rio de Janeiro – CBMERJ - Rio de Janeiro (RJ), Brasil.

**Keywords:** Voz, Qualidade de Voz, Percepção de Fala, Percepção Auditiva, Fonética

## Abstract

**Objetivo:**

Analisar a confiabilidade interavaliadores do Vocal Profile Analysis Scheme – Brazilian Portuguese (VPAS-PB) quando aplicado por juízas experientes.

**Método:**

Dez adultos do sexo masculino, sem queixas vocais ou auditivas, produziram uma frase padrão e dois relatos pessoais. As amostras foram avaliadas de forma independente por três fonoaudiólogas com formação em fonética e experiência prévia com o protocolo. A confiabilidade interavaliadores foi estimada por meio da análise de consistência interna (Alfa de Cronbach).

**Resultados:**

Observou-se consistência interna de boa a excelente para a maioria dos ajustes vocais, com menor concordância nos ajustes “lábios estirados” e “falsete”.

**Conclusão:**

O VPAS-PB apresenta alta confiabilidade interavaliadores e boa consistência interna para a maioria dos ajustes vocais, reforçando sua aplicabilidade no contexto do português brasileiro.

## INTRODUÇÃO

A avaliação perceptivo-auditiva da voz é amplamente reconhecida como uma etapa consolidada na análise da qualidade vocal. Entretanto, seu caráter subjetivo impõe desafios que tornam necessária a adoção de protocolos estruturados, capazes de minimizar a variabilidade entre avaliadores e de orientar descrições sistemáticas dos ajustes vocais observados. Entre esses protocolos, destaca-se o Vocal Profile Analysis Scheme (VPAS), desenvolvido para sistematizar a descrição da voz com base no modelo fonético de qualidade vocal^(1)^.

O modelo fonético de qualidade vocal propõe que a descrição da voz seja feita a partir da observação integrada de pistas auditivas, acústicas, fisiológicas e articulatórias, refletindo as diferentes configurações e funcionamentos do trato vocal durante a fala. O VPAS-PB parte do princípio de que os ajustes vocais ocorrem de forma combinada, o que permite registrar simultaneamente diferentes características da produção vocal. Essa abordagem busca refletir a complexidade real das configurações vocais e ampliar a precisão descritiva da análise perceptiva.

O VPAS foi adaptado para o português brasileiro^(2)^ com o objetivo de manter a estrutura teórica original, mas incorporando ajustes terminológicos que refletissem a realidade linguística e cultural do Brasil. A adaptação levou em conta os avanços na área da fonética e da fisiologia da fala, especialmente no que diz respeito aos aspectos fonatórios. Termos como*breathiness*e*whisper*, por exemplo, foram revisados para garantir maior precisão semântica na equivalência:*whispery voice*passou a ser associada à voz soprosa, enquanto*breathy voice*corresponde à voz murmurada, embora este último termo, comum na fonética descritiva, não tenha sido incluído na tradução final.

Pesquisas nacionais anteriores investigaram a validade da aplicação do VPAS, demonstrando a importância da formação específica dos avaliadores para alcançar níveis adequados de consenso^(3)^. A precisão na identificação dos ajustes vocais depende não apenas do treinamento técnico, mas também da clareza conceitual dos parâmetros que compõem o protocolo.

Estudos internacionais e nacionais reforçam a importância de adotar protocolos estruturados com base no modelo fonético de qualidade vocal, como forma de reduzir a variabilidade entre avaliadores e fortalecer a confiabilidade das análises^(4,5)^. A adoção de referenciais fonéticos claros favorece a avaliação consistente da qualidade vocal.

Além disso, fatores como a qualidade técnica das amostras de áudio (incluindo frequência de amostragem adequada e controle de ruídos) são determinantes para a precisão dos julgamentos perceptivo-auditivos^(6)^.

O treinamento específico dos juízes também exerce influência significativa. Estudos indicam que programas estruturados de treinamento aumentam a concordância entre avaliadores^(7)^, embora revisões de escopo apontem a ausência de consenso sobre a melhor metodologia para formação desses profissionais^(8)^.

A literatura frequentemente utiliza o Índice de Correlação Intraclasse (ICC) para mensurar a confiabilidade entre avaliadores^(9)^, sobretudo em contextos de análise de dados contínuos. No presente estudo, optou-se pelo uso do Alfa de Cronbach, adequado para verificar a consistência interna dos julgamentos categóricos aplicados no VPAS-PB.

Em contextos forenses, a necessidade de avaliações objetivas e reprodutíveis^(6)^ torna ainda mais crítica a adoção de protocolos formalizados. O VPAS-PB, nesse cenário, oferece uma metodologia de avaliação baseada no modelo fonético de qualidade vocal, favorecendo descrições sistemáticas e reprodutíveis da qualidade vocal.

O controle de variáveis sociolinguísticas, fonéticas e vocais tem sido indicado como condição relevante para a validade de análises perceptivo-auditivas^(4)^, e foi incorporado à metodologia deste estudo.

Finalmente, investigações comparativas indicam que o protocolo VPAS em sua versão completa permite descrições mais detalhadas e consistentes em comparação a versões simplificadas^(10)^. Esses achados reforçam a necessidade de ampliar o ensino e a aplicação do modelo fonético de qualidade vocal, por meio do uso do VPAS, para fortalecer a formação de avaliadores e a qualidade das análises em língua portuguesa.

Diante disso, o presente estudo tem como objetivo analisar a consistência interna das avaliações perceptivo-auditivas realizadas com o VPAS-PB, contribuindo para o fortalecimento metodológico das análises de qualidade vocal no português brasileiro.

## MÉTODO

Este é um estudo descritivo observacional, aprovado pelo Comitê de Ética em Pesquisa da instituição (parecer 2.153.565). Todos os participantes assinaram o Termo de Consentimento Livre e Esclarecido (TCLE) previamente à coleta de dados.

Participaram do estudo dez sujeitos adultos, falantes nativos do português brasileiro, com idades entre 34 e 49 anos, residentes na mesma cidade. A escolha por sujeitos do sexo masculino visou controlar variáveis acústicas relacionadas ao sexo, evitando a necessidade de formar dois grupos distintos. A triagem vocal foi realizada por meio de autorrelato e escuta clínica perceptivo-auditiva conduzida pela pesquisadora, a fim de excluir participantes com alterações vocais evidentes. Foram também excluídas pessoas com queixas auditivas, histórico de uso profissional da voz e/ou dificuldades de leitura. Cada participante foi orientado a realizar três gravações de amostras de fala: a leitura de uma frase padrão e dois relatos narrativos espontâneos, nos quais deveriam compartilhar histórias pessoais de impacto emocional. Cada narrativa espontânea teve duração média de um minuto.

As gravações foram realizadas em ambiente controlado, utilizando gravador Zoom H5, com frequência de amostragem de 44.100 Hz, resolução de 16 bits e extensão de arquivo .wav. Os sujeitos foram posicionados a uma distância de 60 centímetros do microfone, a fim de padronizar a captação do sinal sonoro.

As amostras foram organizadas no formato *Collection* no software de livre acesso Praat. Cada conjunto de amostras foi armazenado em pastas individuais, contendo também os respectivos protocolos VPAS-PB a serem preenchidos pelas avaliadoras. Um documento de orientações padronizadas foi incluído junto ao material enviado às juízas.

Três avaliadoras participaram da análise perceptivo-auditiva. Todas possuíam formação em fonética e experiência mínima de quatro anos na aplicação do VPAS-PB (Anexo A). Cada juíza analisou as amostras de forma independente, preenchendo individualmente o protocolo para cada conjunto de amostras de fala. A análise da consistência interna foi realizada com base nesses julgamentos individuais.

Os dados resultantes da avaliação foram organizados em planilhas no software Microsoft Excel e posteriormente submetidos à análise estatística. O índice de consistência interna entre as avaliadoras foi calculado por meio do Alfa de Cronbach, utilizando o software IBM SPSS Statistics, versão 23.0. O nível de significância adotado foi de 5% (p < 0,05).

## RESULTADOS

A análise de consistência interna revelou elevados índices de confiabilidade entre as avaliadoras para a maioria dos ajustes vocais. A maior parte dos valores de Alfa de Cronbach ficou acima de 0,75, indicando consistência considerada boa a excelente. Ajustes específicos apresentaram índices apenas satisfatórios ou insatisfatórios, conforme detalhado na Tabela 1.

**Tabela 1:** Consistência interna entre avaliadoras: Alfa de Cronbach dos ajustes vocais

Ajuste Vocal	Alfa de Cronbach (α)	Significância (p)	Status de Confiabilidade
Lábios arredondados	0,667	0,023	Satisfatório
Lábios estirados	0,585	0,053	Insatisfatório
Labiodentalização	1,000	< 0,001	Elevado
Lábios extensão diminuída	0,892	< 0,001	Elevado
Lábios extensão aumentada	0,893	< 0,001	Elevado
Mandíbula fechada	1,000	< 0,001	Elevado
Mandíbula aberta	0,956	< 0,001	Elevado
Mandíbula extensão diminuída	0,914	< 0,001	Elevado
Mandíbula extensão aumentada	0,881	< 0,001	Elevado
Ponta de língua avançada	0,901	< 0,001	Elevado
Ponta de língua recuada	0,750	0,006	Elevado
Corpo de língua avançado	0,869	< 0,001	Elevado
Corpo de língua recuado	0,654	0,026	Satisfatório
Corpo de língua elevado	0,843	< 0,001	Elevado
Corpo de língua abaixado	0,903	< 0,001	Elevado
Corpo de língua extensão diminuída	0,686	0,018	Satisfatório
Constrição faríngea	0,747	0,006	Elevado
Expansão faríngea	0,960	< 0,001	Elevado
Escape nasal audível	1,000	< 0,001	Elevado
Nasal	1,000	< 0,001	Elevado
Denasal	1,000	< 0,001	Elevado
Laringe elevada	0,853	< 0,001	Elevado
Laringe abaixada	0,780	0,003	Elevado
Hiperfunção de trato	0,862	< 0,001	Elevado
Hipofunção de trato	1,000	< 0,001	Elevado
Hiperfunção laríngea	0,956	< 0,001	Elevado
Falsete	0,585	0,053	Insatisfatório
Crepitância	1,000	< 0,001	Elevado
Voz crepitante	0,852	< 0,001	Elevado
Escape de ar	1,000	0,001	Elevado
Voz soprosa	0,667	0,023	Satisfatório
Voz áspera	0,932	< 0,001	Elevado
Pitch habitual elevado	0,821	0,001	Elevado
Pitch habitual abaixado	1,000	< 0,001	Elevado
Pitch extensão diminuída	0,750	0,006	Elevado
Pitch variabilidade diminuída	0,646	0,029	Satisfatório
Pitch variabilidade aumentada	1,000	< 0,001	Elevado
Loudness habitual aumentado	1,000	< 0,001	Elevado
Loudness habitual diminuído	0,926	< 0,001	Elevado
Loudness extensão aumentada	1,000	< 0,001	Elevado
Loudness extensão diminuída	1,000	< 0,001	Elevado
Loudness variabilidade aumentada	1,000	0,001	Elevado
Loudness variabilidade diminuída	1,000	< 0,001	Elevado
Continuidade interrompida	1,000	< 0,001	Elevado
Taxa de elocução rápida	0,892	< 0,001	Elevado
Taxa de elocução lenta	1,000	< 0,001	Elevado
Suporte respiratório adequado	1,000	< 0,001	Elevado
Suporte respiratório inadequado	1,000	< 0,001	Elevado

**Legenda:** α = Alfa de Cronbach. Testes estatísticos utilizados: Análise de Consistência Interna

## DISCUSSÃO

Os resultados deste estudo mostraram que a aplicação do VPAS-PB nas avaliações perceptivo-auditivas da qualidade vocal gerou altos índices de consistência interna entre as avaliadoras, indicando a confiabilidade do protocolo no contexto do português brasileiro. O Alfa de Cronbach revelou valores elevados, com a maioria dos ajustes vocais apresentando consistência muito boa, conforme detalhado na Tabela 1.

A consistência foi particularmente alta para ajustes de tensão laríngea e supraglóticos, com valores de Alfa de Cronbach superiores a 0,90, refletindo a clareza com que esses ajustes são percebidos pelas avaliadoras. O modelo fonético de qualidade vocal facilita a identificação de ajustes mais evidentes na fala^(1-3,10)^, o que contribui para a alta confiabilidade observada nesses casos.

Por outro lado, alguns ajustes, como lábios arredondados, corpo de língua recuado e*pitch* variabilidade diminuída, apresentaram consistência interna considerada apenas satisfatória (Alfa de Cronbach entre 0,60 e 0,70). Esses ajustes envolvem características mais sutis e difíceis de identificar, o que é consistente com a literatura, que aponta maior variação perceptiva em parâmetros de maior saliência auditiva^(3,11)^.

Dois ajustes (lábios estirados e falsete) apresentaram consistência interna insatisfatória (Alfa de Cronbach abaixo de 0,60). Esse resultado indica maior variabilidade entre as avaliadoras, possivelmente devido à dificuldade em identificar e classificar esses ajustes de maneira consistente. A literatura também destaca que ajustes sutis ou com baixa saliência perceptiva tendem a apresentar menor confiabilidade interavaliadores^(5)^.

Os resultados demonstraram que, embora o VPAS-PB seja um protocolo robusto, ajustes relacionados a características mais sutis ou difíceis de discriminar, como lábios estirados ou falsete, apresentaram menor concordância. Uma possível explicação é que tais ajustes podem ter sido pouco utilizados pelos participantes ou não suficientemente marcados para serem percebidos com clareza. Considera-se ainda a possibilidade de que, por se tratarem de vozes masculinas, certos ajustes tenham sido evitados ou pouco explorados. Um estudo anterior identificou diferenças no uso de ajustes vocais entre homens e mulheres^(12)^. Além disso, fatores sociolinguísticos podem ter contribuído para a menor ocorrência desses ajustes, dificultando sua detecção.

Este estudo focou na consistência interna das avaliações, um aspecto importante para verificar a reprodutibilidade do protocolo em diferentes contextos e com diferentes avaliadores. O uso do Alfa de Cronbach foi adequado para medir a confiabilidade das avaliações realizadas, uma vez que o objetivo não era mensurar a variabilidade entre os sujeitos, mas sim a homogeneidade interna das avaliações.

Os achados também indicam que, apesar da boa confiabilidade geral, a formação contínua das avaliadoras é um fator decisivo para melhorar a consistência nos julgamentos de ajustes de menor saliência perceptiva. Estudos recentes têm apontado a importância de programas de treinamento mais detalhados em avaliações perceptivo-auditivas^(8)^, especialmente frente às discussões sobre a suposta subjetividade desse tipo de análise^(13)^. Embora haja críticas à precisão de análises baseadas exclusivamente na percepção auditiva, também existem questionamentos à ideia de que apenas os métodos instrumentais seriam objetivos, uma vez que sua aplicação envolve decisões humanas e, portanto, está igualmente sujeita a variabilidade^(11)^.

Além do treinamento, considera-se que a alta confiabilidade observada neste estudo também possa estar relacionada à experiência prévia das avaliadoras e à continuidade no uso do VPAS-PB ao longo do tempo. A familiaridade com o protocolo, desenvolvida por meio de sua aplicação prática recorrente, pode ter favorecido a estabilidade dos julgamentos, especialmente nos ajustes mais sutis^(3)^. Esse fator, embora não tenha sido mensurado diretamente, deve ser considerado em futuras investigações sobre o impacto da experiência acumulada na confiabilidade perceptiva.

Apesar da ampla aplicabilidade do VPAS-PB em contextos clínicos e de pesquisa^(14,15)^, estudos anteriores concentram-se majoritariamente em grupos restritos quanto a sexo, faixa etária e variedade linguística^(12)^. A maioria das investigações envolve falantes adultos do sexo feminino, muitas vezes do mesmo recorte regional e sociolinguístico, o que pode limitar a generalização dos achados. Assim, recomenda-se que pesquisas futuras explorem a aplicação do protocolo em populações mais diversas, incluindo diferentes gêneros, faixas etárias, sotaques regionais e línguas, a fim de verificar se tais variáveis influenciam a percepção e a ocorrência dos ajustes descritos, contribuindo para a ampliação da validade ecológica do instrumento, incluindo o uso em perícias^(10,11)^.

Em contextos forenses, a precisão das avaliações perceptivo-auditivas é especialmente relevante, e o VPAS-PB oferece uma abordagem robusta para garantir descrições detalhadas e consistentes da qualidade vocal, minimizando a subjetividade nas avaliações^(10)^, o que reforça a eficácia do modelo fonético de qualidade vocal adaptado ao português brasileiro.

## CONCLUSÃO

O presente estudo demonstrou que o VPAS-PB apresenta alta confiabilidade interavaliadores, com consistência interna excelente na maioria dos ajustes vocais. Esses achados reforçam sua aplicabilidade no contexto do português brasileiro. No entanto, ajustes com menor saliência perceptiva, como lábios estirados e falsete, revelaram menor concordância, apontando a necessidade de formação continuada dos avaliadores.

A utilização do Alfa de Cronbach mostrou-se eficaz para aferir a homogeneidade dos julgamentos, e os resultados sugerem que a familiaridade com o protocolo pode favorecer a estabilidade das análises.

Futuros estudos devem ampliar a diversidade amostral e investigar o impacto de variáveis sociolinguísticas, fonéticas e contextuais, incluindo aplicações em perícias. O VPAS-PB se mostra promissor como ferramenta sistemática para avaliações vocais confiáveis e replicáveis.

## Anexo A. Protocolo VPAS-PB

**Table:**

	**PRIMEIRA PASSADA**		**SEGUNDA PASSADA**									
	**Neutro**	**Não neutro**					**Moderado**			**Extremo**		
							**1**	**2**	**3**	**4**	**5**	**6**
**1. Lábios**	**Neutro**	**Não neutro**	**Protraídos**									
			**Estirados**									
			**Labiodentalização**									
			**Extensão diminuída**									
			**Extensão aumentada**									
**2. Mandíbula**	**Neutro**	**Não neutro**	**Fechada**									
			**Aberta**									
			**Protraída**									
			**Extensão diminuída**									
			**Extensão aumentada**									
**3. Língua ponta/lâmina**	**Neutro**	**Não neutro**	**Avançado**									
			**Recuado**									
**4. Corpo de língua**	**Neutro**	**Não neutro**	**Avançado**									
			**Recuado**									
			**Elevado**									
			**Abaixado**									
			**Extensão diminuída**									
			**Extensão aumentada**									
**5. Faringe**	**Neutro**	**Não neutro**	**Constrição**									
			**Expansão**									
**6. Velofaringe**	**Neutro**	**Não neutro**	**Escape nasal audível**									
			**Nasal**									
			**Denasal**									
**7. Altura de laringe**	**Neutro**	**Não neutro**	**Elevada**									
			**Abaixada**									
**B. TENSÃO MUSCULAR GERAL**												
**8. Tensão de trato vocal**	**Neutro**	**Não neutro**	**Hiperfunção**									
			**Hipofunção**									
**9. Tensão laríngea**	**Neutro**	**Não neutro**	**Hiperfunção**									
			**Hipofunção**									
**C. ELEMENTOS FONATÓRIOS**												
	**AJUSTE**			**Presente**			**Graus de Escala**					
				**Neutro**		**Não neutro**	**Moderado**			**Extremo**		
							**1**	**2**	**3**	**1**	**2**	**3**
**10. Modo de fonação**	**Modal**											
	**Falsete**											
	**Crepitância/ *vocal fry***											
	**Voz crepitante**											
**11. Fricção laríngea**	**Escape de ar**											
	**Voz soprosa**											
**12. Irregularidade Laríngea**	**Voz áspera**											
**DINÂMICA VOCAL**		**Neutro**	**AJUSTE**				**Moderado**			**Extremo**		
							**1**	**2**	**3**	**1**	**2**	**3**
**D. ELEMENTOS PROSÓDICOS**												
**13. Pitch (f0)**		**Habitual**	**Diminuída**									
			**Aumentada**									
		**Extensão**	**Diminuída**									
			**Aumentada**									
		**Variabilidade**	**Diminuída**									
			**Aumentada**									
**14. Loudness (intensidade)**		**Habitual**	**Diminuída**									
			**Aumentada**									
		**Extensão**	**Diminuída**									
			**Aumentada**									
		**Variabilidade**	**Diminuída**									
			**Aumentada**									
**15. TEMPO**												
**Continuidade**					**Interrompida**							
**Taxa de elocução**					**Rápida**							
					**Lenta**							
**16. OUTROS ELEMENTOS**												
**Suporte Respiratório**					**Adequado**							
					**Inadequado**							

**Legenda:** Protocolo Vocal Profile Analysis Scheme (adaptado para o Português Brasileiro por Camargo e Madureira, 2008).
